# Remarks of Dr. C. W. Barrett upon Dental Literature

**Published:** 1882-12

**Authors:** 


### Remarks of Dr. W. C. Barrett upon Dental Literature.

[Before the New England Dental Society, Boston, Oct. 6, 1882.

I am asked to say something about the staff upon which our profession leans. Without a literature, men are barbarians. Without a literature, dentistry degenerates into mere hand-craft. Before the day in which Cadmus invented letters, Greece was the home of petty tribes of savages. What distinguished and made forever memorable that early Egyptian people, with their wondrous civilization, but the fact that they, of all their contemporaries, had a written language—aye—three written languages? What marked the golden age of Pericles, of Solon, of Socrates, of Thucydides, but the high tide of literature? What were the palmy days of Rome but those of Virgil, and Pliny, and Cicero, and Horace? Where were the fame of Agamemnon, and Achilles, and Ajax, and Nestor, but for the fact that Homer lived? And to come down to more modern times, what were the golden days of England but those of Bacon, and Addison, and Milton, and Shakespeare? Nay, to strike yet nearer home, what names have thrown greater luster upon American history than those of her literary men; her Longfellows, and Irvings, and Hawthornes, and Whittiers, who shall be known when her warriors, the successful murderers of their fellow-men, shall have sunk into oblivion?

Dentistry has made wonderful progress in the past, because we have had a class of writers who, knowing what they desired to say, had the happy faculty of expressing it tersely, impressively. The pen of Harris was more powerful than his probe, or excavator. McQuillen made a deeper impression upon the minds of his fellow dentists, than he did upon his patients. Why need I go on to mention Bond, Taft, Arthur, and others? The sum of the whole matter is, we get a clear picture of dentistry by looking in the mirror of its literature. Every feature is reflected therein, and when our letters are at a low ebb, we may be sure the profession is not above the same level. I will not extenuate, nor set down aught in malice. We have a few pretty fair text-books, but not half as many as we should have. If one desires to write up any subject in dentistry, when he looks about him for his authorities and quotations he is quickly reminded of a want which cries aloud. We have a fine array of dental journals, and they are a fair representation of the profession, but with two exceptions, possibly three, they are owned and practically controlled by the mercantile interest in dentistry, and are mainly published as advertising mediums.

The part of each which is devoted to the profession is not what it would be if dentists were more communicative, but it is pretty well as compared with other professions. Our journals are in the main not very deep, but they are broad. It is a shame to us of the rank and file, that so many articles are necessarily admitted as padding—to fill up. There is a great deal of borrowing from each other, and not enough of original essays and primary thought. How can there be, when dentists are so backward in preparing articles—and when the journals pay nothing for the better class of writers, and must, therefore, put up with what they can get? There is a deal of red rubber and amalgam talk, and not enough of basal principles, but the journals are struggling toward higher ground. The old seniors, like the REGISTER, the *American Journal*, and the *Cosmos*, hold their own fairly well among the younger ones, though the indications in the juniors of young blood, later investigation, and fresh thought, it may be plainly seen, nettle the old ones at times. Altogether we have no reason to blush very deeply for our literature. It would be better if there were less of dental depot influence (I do not intimate dictation), for the light is not becoming more manifest when, practically, the depots openly combine against the dentists, and not a journal raises the voice of warning, but in the main our periodicals are as unshackled as, in the nature of things, they well can be.

What is the outlook for the future? That depends upon the progress of the profession. If dentists are thinking, reflecting men our muses will show it. If the profession is everywhere given up to the "auri sacra fames," the cursed thirst for gold, we shall soon have cause to hang our heads for our humanities, because the stream can not rise higher than its source, and professional literature will remain at the level of the body with which it is connected.

It must be remembered that every profession makes its own literature. Editors write few of the articles which they publish. Look through the list of our journals, and see what proportion is conducted by practical, working dentists. The busy bees of the profession have little time for composing homilies upon the flowers which they are engaged in rifling of their honey. But if practical dentists neglect the journals altogether, we shall soon see them what some of the more prominent foreign dental periodicals are to-day—filled up with accounts of the success of the dentists in crowding themselves into medicine, and exhibiting their inability to stand alone; with long narratives of legal squabbles; the platitudes of mutual admiration societies; of meetings where a paragraph suffices to record all the scientific discussions, while pages are devoted to an account of the dinner; ten lines dedicated to the brain, ten pages to the stomach. I like good eating and drinking, but my God is *not* my belly, however much appearances may be against me.

The Literature of Dentistry, let me repeat, can only reflect the status of the profession. If dentists are wholly given up to greed and money-making, the journals will be devoted to advertising. Hah! I almost wish I had not said that, for it reminds me that I counted the pages of the last dental journal received before leaving home, and—let me say it in a whisper—there was more space devoted to advertising than to dental literature. It was a depot journal, however. I will say for the credit of the only dental journal which New England possesses, that I did the same for its September number, and found the reading matter was more than four times the advertisements; so I conclude that is not mainly published as an advertising sheet. Well, advertisements are legitimate enough, but I don't like to see our very altars inscribed—"Try Dr. Quack's new patent, improved, non-shrinkable, Stannous gold, New Departure alloy; in proportion as teeth need saving, amalgam is the only material proper to save them, and mine is the single, solitary, genuine, Simon-pure, Old Original Jacob Townsend, take-no-other-only-four-dollars-an-ounce-article." Such literature as that, whether in book or journals, private conversation or dental society discussions, is scarcely creditable to the profession, and those who last summer attended the International Medical Congress in London had it frequently thrown in their teeth.

The only way to sustain a creditable literature is for every intelligent dentist to contribute his mite. There is not a practitioner worthy the name, who has not stored in his memory some aphorism that others need to be reminded of; whose experience, in some one instance peculiar to himself and unique, should not illumine and instruct his brethren. There are a few gifted men who might, perhaps, contribute volumes. Not one of us but could enrich our literature with a paragraph, at least. A thought which may occur to us, if properly recorded, if garnered in the grand storehouse of letters, may feed some hungry soul, and stimulate a fainting mind to renewed exertion. Neglected, it is as the idle wind which fanned the brow of yesterday, and to-day is not. Brethren, don't forget the dental journals.

